# Investigation of geo-spatial hotspots for the occurrence of tuberculosis in Almora district, India, using GIS and spatial scan statistic

**DOI:** 10.1186/1476-072X-5-33

**Published:** 2006-08-10

**Authors:** Neeraj Tiwari, CMS Adhikari, Ajoy Tewari, Vineeta Kandpal

**Affiliations:** 1 Department of Statistics, Kumaon University, S.S.J.Campus, Almora, Uttaranchal, India; 2 PCI Software Pvt. Ltd., New Delhi, India; 3 Community Health Centre, Sidhauli, U.P., India; 4 Department of Anatomy, GSVM Medical College, Kanpur, U.P., India

## Abstract

**Background:**

The World Health Organization has declared tuberculosis a global emergency in 1993. It has been estimated that one third of the world population is infected with *Mycobacterium tuberculosis*, the causative agent of tuberculosis. The emergence of TB/HIV co-infection poses an additional challenge for the control of tuberculosis throughout the world. The World Health Organization is supporting many developing countries to eradicate tuberculosis. It is an agony that one fifth of the tuberculosis patients worldwide are in India. The eradication of tuberculosis is the greatest public health challenge for this developing country. The aim of the present population based study on *Mycobacterium tuberculosis *is to test a large set of tuberculosis cases for the presence of statistically significant geographical clusters. A spatial scan statistic is used to identify purely spatial and space-time clusters of tuberculosis.

**Results:**

Significant (p < 0.05 for primary clusters and p < 0.1 for secondary clusters) high rate spatial and space-time clusters were identified in three areas of the district.

**Conclusion:**

There is sufficient evidence about the existence of statistically significant tuberculosis clusters in Almora district of Uttaranchal, India. The spatial scan statistics methodology used in this study has a potential use in surveillance of tuberculosis for detecting the true clusters of the disease.

## Background

Tuberculosis (TB) is an infectious disease caused by the bacillus *Mycobacterium tuberculosis *and spreads through air by a person suffering from TB. The 1990 World Health Organization (WHO) report on the Global Burden of Disease ranked TB as the seventh most morbidity-causing disease in the world, and expected it to continue in the same position up to 2020 [1]. In 2001, the WHO estimated that 1.86 billion persons (32% of the world population) were infected with TB. Each year, 8.74 million people develop TB and nearly 2 million die. This means that someone somewhere contracts TB every four seconds and one of them dies every 10 seconds [2,3]. Unless properly treated, an infectious pulmonary TB (i.e., the TB of lungs) patient can infect 10–15 people in a year [4]. TB kills more adults than any other infectious disease worldwide, accounting for almost 400,000 deaths annually [4]. It mainly afflicts people who are in the economically productive years of their life (15–54 years), thereby causing large social and economic burden on a country [4]. This, in turn, hampers the development of a country. Another cause of concern is the finding that HIV (Human Immuno-deficiency Virus) infection has a marked impact on the progression of TB as the two diseases are closely linked. TB is the most common opportunistic disease that affects people infected with HIV. As HIV debilitates the immune system, vulnerability of TB is increased manifold. It is estimated that without HIV, the lifetime risk of TB-infected people developing tuberculosis is only 10%, compared to over 50% in the case of people co-infected with HIV and TB [5,6]. HIV is also the most powerful risk factor for the progression of TB-infection to the disease. In a reciprocal manner, TB accelerates the progression of HIV into AIDS (Acquired Immune Deficiency Syndrome), thus shortening the survival of patients with HIV infection. Fortunately, TB is a curable disease even among the HIV-infected people. The prevalence of TB and HIV co-infection worldwide is 0.18% and about 8% TB cases have HIV infection [3].

According to the WHO Report 2004 on Global TB Control, India is sharing 20% burden of TB patients worldwide, and is leading the 22 high burden countries in the world. The estimated number of TB cases in India is 42.26 million (44% of the total population) with 1.8 million people developing TB every year and nearly 0.5 million dying annually due to it [7]. More than 1,000 people in a day and one in every minute die of TB in India [8]. In India, Every year 300,000 children leave their schools due to this ghastly disease. Economic burden to the society is to the tune of $ 3 billion [4]. The emergence of TB/HIV co-infection poses an additional challenge to the control of TB in India. According to the 2006 Report on Global AIDS Epidemic, India has the highest number of people infected with HIV. It has 5.7 million HIV infected persons by the end of 2005, against 5.5 million in South Africa [9]. While TB/HIV co-infection rates are highest in Africa, more people (approximately 2 million) are co-infected in India than in any other country [3]. Moreover, HIV is beginning to make its forays in rural areas of high prevalence states. The spread of HIV and Multi-drug Resistant Tuberculosis (MRT) asks for urgent action from health authorities.

With such a magnitude of disease and looming danger of HIV co-infection, TB is the biggest public health challenge for India. An adult suffering from TB loses on an average three to four months of working time. This translates to a loss of 20–30% of household's annual income [4]. The burden of TB is enormous but is hidden by stigma and poor diagnostic quality (i.e., the microscopic examination of sputum is rarely done because of non-availability of trained staff and microscopes, and hence there is an over-dependence on X-ray reports for diagnosis). Women with TB are often severely stigmatised. A recent study in India suggests that more than 100,000 women are rejected by their families each year on account of TB [4]. TB kills more women than all causes of maternal mortality combined. It also adversely affects child-care. A substantial proportion of female infertility cases are also caused by TB.

India spends only 4.5% of GDP on health. The public sector health expenditure is 0.9% of GDP and less than 10% of Indians have access to any health insurance [10]. The current annual per capita public health expenditure in the country is no more than Rupees 200 [11]. The social and economic ramifications of this disease in India can be assessed from these facts.

To contain this scourge, the National Tuberculosis Control Program (NTCP) was adopted in India in 1962. However, the desired results were not forthcoming. There was over-dependence on X-ray for diagnosis. Treatment regimens used were often non-standard and incomplete treatment was the norm rather than an exception.

On the recommendations of an expert committee in 1992, a revised strategy known as Revised National Tuberculosis Control Program (RNTCP) was adopted to control the spread of TB in India. The WHO is supporting this program. It was pilot-tested in 1993 in a population of 2.35 million and was then extended to a population of 13.85 million in 15 States/Union Territories of the country [8]. Rapid RNTCP expansion began in late 1998. By September 2005, around 95% (about 1,059 million) of the population has been covered under RNTCP [8]. In India, every day more than 15,000 patients are being examined for TB free of charge under RNTCP [8]. The goal of RNTCP is to cure at least 85% of new sputum-positive patients after achieving the target of detecting 70% of the newly infected sputum-smear-positive cases. It is expected that under the RNTCP, every year 1,350 patients per million population will be cured of TB. These targets are achievable by the application of DOTS (Directly Observed Treatment with Short course chemotherapy).

The basic problems in geographical surveillance for a spatially distributed disease are the identification of areas of exceptionally high prevalence, to test their statistical significance, and to identify the reasons behind the elevated prevalence of the disease. A hotspot is an area of high response or an elevated cluster for an event. Temporal, spatial, and space-time scan statistics are commonly used for disease cluster detection and evaluation. Some of them are either able to detect clusters with no inference involved, or they do inference without the ability to detect the location of clusters. However, the spatial scan statistic developed by Martin Kulldorff [12] can both detect and provide inference for spatial and space-time disease clusters. The spatial scan statistic implemented in SaTScan software [13] offers several advantages over the existing techniques for detection of disease clusters. Temporal, spatial and space-time scan statistics [12,14-16] are now commonly used for disease cluster detection and evaluation, for many diseases including cancer [17-22], Creutzfeldt-Jakob disease [23], granulocytic ehrlichiosis [24], sclerosis [25], diabetes [26], and giardiasis [27]. There had been no studies to detect the statistically significant clusters of TB in Uttaranchal, India. The detection of these clusters may be highly useful in surveillance of the disease, finding the factors behind the spread of the disease, and making suitable policies to control these factors.

Using spatial scan statistic and GIS, we have investigated the spatial distribution of confirmed cases of TB and identified the areas of high risk within the boundaries of Almora district of Uttaranchal, India. In this study we have used the geographical information systems (GIS) and a spatial scan statistic to investigate statistically significant hotspots of TB in Almora district of Uttaranchal during 2003–2005. The tools used in this study provide an opportunity to clarify and quantify the health burden of TB in this hilly region of Uttaranchal.

## Results

### Purely spatial analysis

Using the maximum spatial cluster size of ≤ 50% of the total population, the spatial cluster analysis identified the most likely significant cluster for high occurrence of TB at DTC, Almora. The overall relative risk (RR) within the cluster was 4.042 with an observed number of 455 cases treated during 2003–2005, compared with 142.40 expected cases. A statistically significant secondary cluster for high occurrence of TB was also detected at Chaukhutiya with RR 1.648, observed cases 118, and expected cases 73.71.

**Figure 1:**
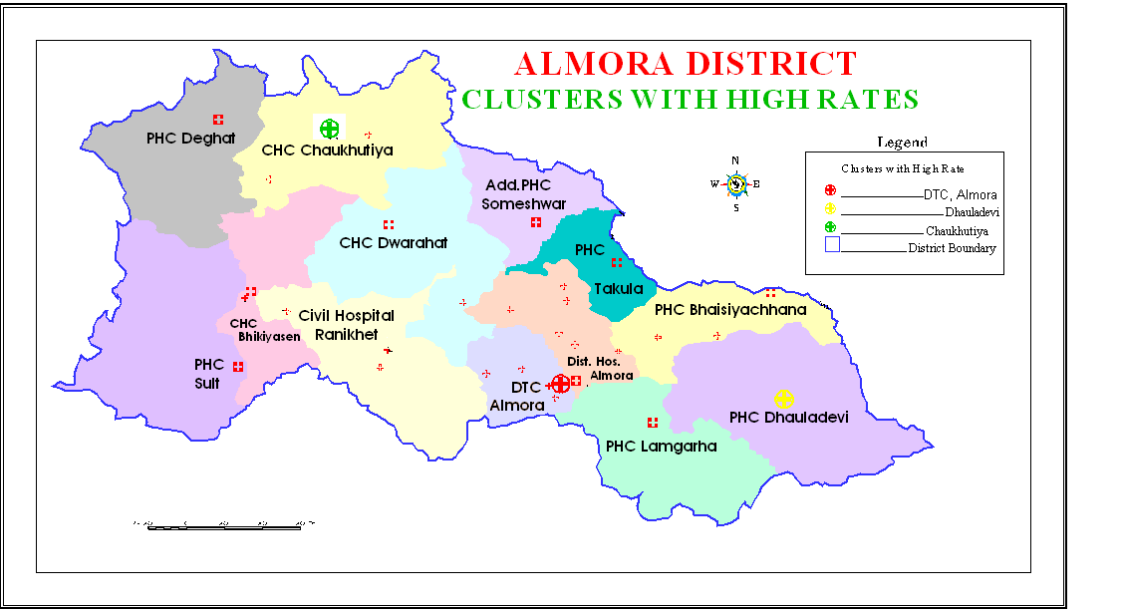
**Spatial distribution of significant high rate tuberculosis clusters in Almora district, India**. The three statistically significant clusters detected by the purely spatial and retrospective space-time analyses with a maximum cluster size of 25% of the total population have been presented in this map.

**Figure 2:**
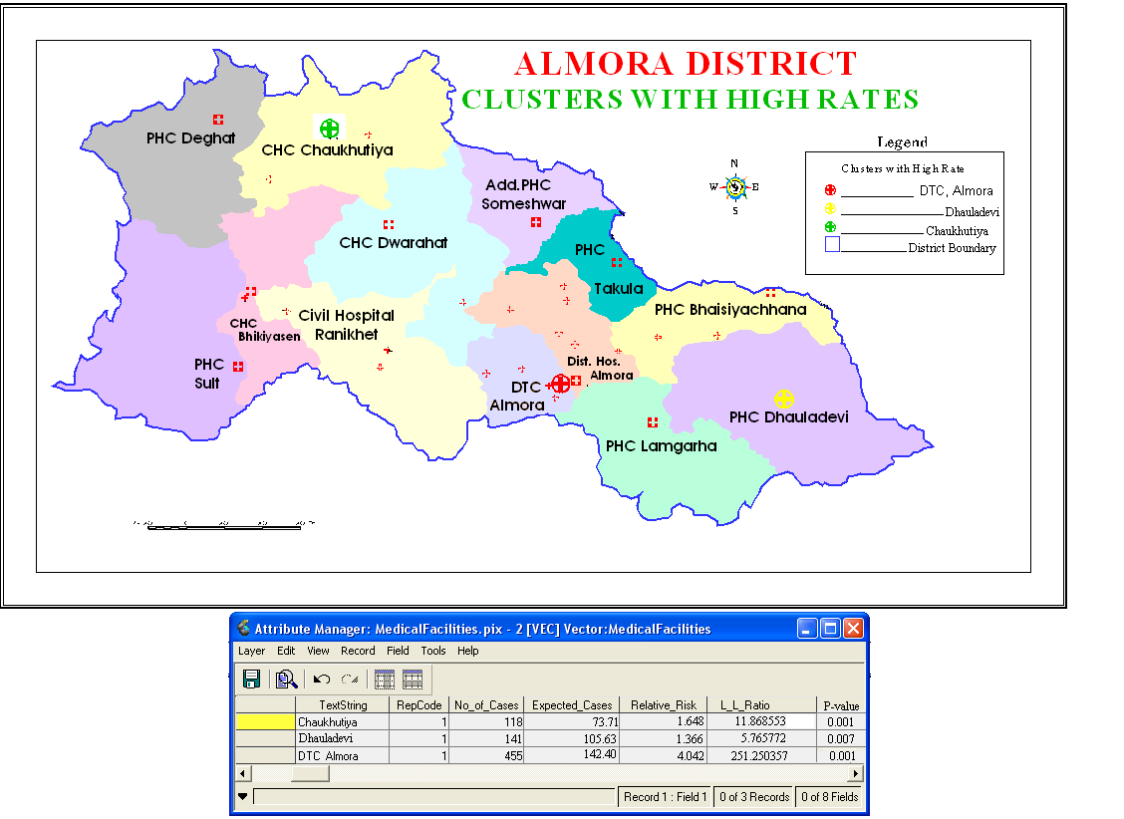
**Significant purely spatial high rate tuberculosis clusters in Almora district, India with relevant database attached**. In this map, the database given in Table 1 is attached to three statistically significant clusters detected by the purely spatial analysis with a maximum cluster size of 25% of the total population.

To investigate the possibility of smaller clusters, when the same analysis was performed with a maximum spatial cluster size of ≤ 25% of the total population, three statistically significant clusters were identified. Out of these three identified clusters, two were the same at DTC Almora and Chaukhutiya detected in the earlier analysis whereas a new statistically significant secondary cluster for high occurrence of TB emerged at Dhauladevi, with RR 1.366 (p = 0.007), observed cases 141, and expected cases 105.63. The observed count, expected count, relative risk, log likelihood, and p-value for each area of excess proportion of TB are presented in Table 1. These three clusters having high rates of TB patients are highlighted in Figure 1, and the database shown in Table 1 is attached to the three clusters in Figure 2.

**Table 1:** Significant high rate spatial tuberculosis clusters in Almora district, India, detected by purely spatial analysis. This table depicts the results of a purely spatial analysis with a maximum spatial cluster size of ≤ 25% of the total population in Almora district, India, during 2003–2005

Cluster with location ID	No. Of Cases	Expected Cases	Relative Risk	Log Likelihood Ratio	P-value
Most Likely Cluster					
1. DTC Almora	455	142.40	4.042	251.250357	0.001
Secondary Clusters					
2. Chaukhutiya	118	73.71	1.648	11.868553	0.001
3. Dhauladevi	141	105.63	1.366	5.765772	0.007

### Space-time analyses

The results of the space-time analysis are somewhat similar to the purely spatial analysis in that the areas of excess incidence of TB from the purely spatial analysis were also statistically significantly high in the space-time analysis. Using a spatial window that could include up to 50% of population at risk and a maximum temporal window of 50% without including purely spatial clusters, the most likely statistically significant cluster for high occurrence of TB was again found to exist at DTC, Almora for the year 2004 (RR = 4.547, p = 0.001), with 196 observed cases and 47.55 expected cases. One statistically significant secondary cluster was also detected at Chaukhutiya for 2003 (RR = 1.682, p = 0.091), with 40 observed cases and 24.02 expected cases.

**Figure 3:**
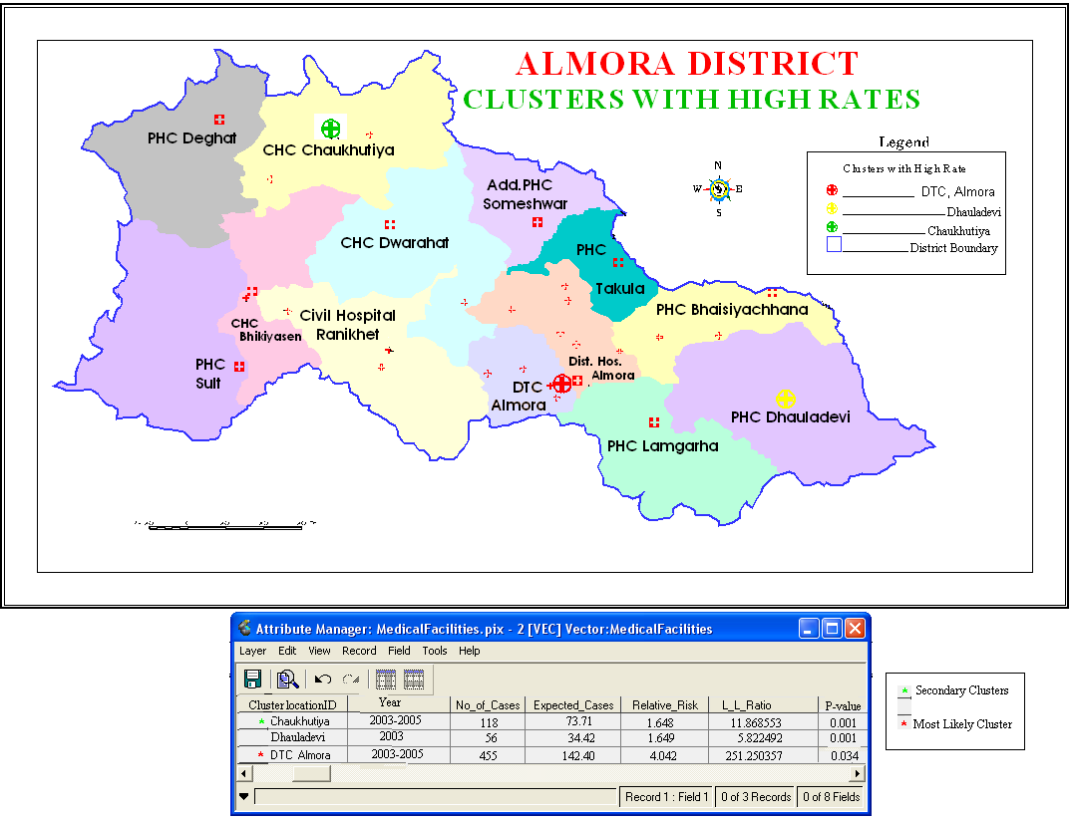
**Significant space-time high rate tuberculosis clusters in Almora district, India with relevant database attached**. In this map, the database given in Table 2 is attached to three statistically significant clusters detected by the retrospective space-time analysis with a maximum cluster size of 25% of the total population.

The results of the space-time analysis using a maximum temporal window of 90% (which included purely spatial clusters as well) and a spatial window that could include up to 25% of the population at risk (which included purely temporal clusters also) are presented in Table 2. From Table 2, we observe that the most likely statistically significant cluster for high occurrence of TB was again found to exist at DTC, Almora for the year 2003–2005. Two statistically significant secondary clusters were also detected for high occurrence of TB, existing at Chaukhutiya for 2003–2005 and Dhauladevi for 2003. This shows that the two statistically significant clusters from the purely spatial analysis, DTC Almora and Chaukhutiya, remained statistically significant for the whole three-year study period. However, the third significant cluster located at Dhauladevi was significant only for the year 2003, with RR 1.649 (p = 0.034), 56 observed cases, and 34.42 expected cases. The database given in Table 2 is attached to the three clusters in Figure 3.

**Table 2:** Significant high rate spatial tuberculosis clusters in Almora district, India, detected by retrospective space-time analysis. This table depicts the results of the space-time analysis using a maximum temporal window of 90%, which included purely spatial clusters as well, and a spatial window of ≤ 25% of the population at risk, which included purely temporal clusters also, in Almora district, India, during 2003–2005

Cluster with location ID	Year	No. Of Cases	Expectd Cases	Relative Risk	Log Likelihood Ratio	P-value
Most Likely Cluster						
1. DTC Almora	2003–2005	455	142.40	4.042	251.250357	0.001
Secondary Clusters						
2. Chaukhutiya	2003–2005	118	73.71	1.648	11.868553	0.001
3. Dhauladevi	2003	56	34.42	1.649	5.822492	0.034

## Discussion

Cluster analyses are important in epidemiology in order to detect aggregation of disease cases, to test the occurrence of any statistically significant clusters, and ultimately to find evidences of etiologic factors. Cluster analysis identifies whether geographically grouped cases of disease can be explained by chance or are statistically significant. It detects true clusters of disease from cases grouped around population centres.

The use of GIS with spatial statistics including spatial filtering and cluster analysis has been applied to many diseases to analyse and more clearly display the spatial patterns of these diseases [28-33]. Spatial scan statistic [12] implemented in SaTScan software [13] is being widely used to detect the clusters of different diseases worldwide [17-27]. Multiple molecular techniques in conjunction with surveillance data and GIS have been used by researchers for detecting the clusters of TB and identifying the outbreaks of *Mycobacterium tuberculosis *[34-37]. A nationwide disease survey conducted by the Indian Council of Medical Research (ICMR) in 1955–1958 provided for the first time an insight into the enormity of the problem in India [38]. Most epidemiological studies on TB subsequent to the ICMR disease survey have been conducted in smaller geographical areas, mostly in southern part of the country. To assess the prevailing epidemiological situation of TB in India, a nationwide tuberculin survey was conducted during 2000–2003 [39-42]. However, the scan statistic has been used for the first time to detect the statistically significant clusters of TB in this study.

The results of the study suggest that there are statistically significant hotspots of TB in three areas of the district. Both purely spatial analysis as well as retrospective space-time analysis showed the existence of TB clusters at the same geographical areas of the district. The most likely cluster was detected at DTC, Almora. Two statistically significant secondary clusters at Chaukhutiya and Dhauladevi were also detected in the two analyses. When the space-time analysis was modified to find either 1,2 or 3-year length clusters, two clusters from the space-time analysis (viz., DTC Almora and Chaukhutiya) became purely spatial clusters whereas the third cluster located at Dhauladevi remained a space-time cluster for the year 2003 only. This provides some evidence that the two statistically significant clusters at DTC, Almora, and Chaukhutiya persist over time whereas there are important temporal differences in the case of the statistically significant cluster detected at Dhauladevi.

The result of the present study provides useful information on the prevailing epidemiological situation of tuberculosis in Almora district of Uttaranchal. The new knowledge about the presence of hotspots of TB in the district can help the district tuberculosis units to intensify their remedial measures in the identified areas of high tuberculosis prevalence and chalk out future strategies for more effective TB control. DTC, Almora was identified the most probable region for the occurrence of TB in the district. The district health authorities should focus more seriously in this microscopic centre (MC) to control the spread of TB in the district. In particular, vigorous efforts are required to intensify the case finding activities in these three TB-infested areas of the district. Compulsory BCG immunization of the children, better coordination of government and private sector, further promotion of general health and hygiene, and improvement in nutritional status of the community, in DTC, Almora, and the other two hotspots detected by the study, can lead to control of TB in the district. Further, it has been found that the hospitals of good repute get sizeable number of patients from other tuberculosis units (TU). The District Tuberculosis Centres (DTC) must work to activate all TUs by posting adequate staff to non-active TUs and providing them better facilities. One limitation of DOTS program is that it is more hospital-centric, laying more emphasis on diagnosis and treatment, and less on case finding and public awareness at community level. Our study can serve as a bridge to fill this gap between the providers and beneficiaries. This strategy can be highly useful in eradication of TB from the country.

The present study has some potential limitations, too. It is not a survey-based study but sentinel surveillance, as the patients presenting themselves in the hospital are only being taken into account. This is so because DOTS program on which this study is based is also a hospital-centric program. Age, sex, religion and other socio-economic and environmental factors are not taken into account due to non-availability of appropriate records of these factors in the hospitals from where secondary data for the study was collected. Some potential variables, such as differential TB reporting in different areas, biased census numbers and covariates that could not be adjusted for, could bias the results of the study. Popular hospitals with good track record attract more patients from nearby areas and this could influence the results of the study. The present study was conducted at the level of thirteen MCs working in the district, which are concerned with the sputum examination (i.e., the diagnosis) of the patients. This can be further extended to the level of treatment centres (TC) and DOTS providers at the village level, to give a more realistic distribution of the disease.

The present study only analyses the statistically significant clusters of TB in Almora district of Uttaranchal. Future research could focus on the effect of various socio-economic and environmental factors on the high occurrence of TB in the hilly region of the state. It is an established fact that the incidence of TB increases with age. There are several other risk factors responsible for the disease such as, cigarette smoking, alcohol abuse, injection drug use, and malnutrition [43]. They adversely affect immune system, and so could enhance the TB incidence. The factors mainly responsible for high occurrence of TB in hilly region of Uttaranchal may be attributable to the poor socio-economic conditions of the people, poor nutritional status, smoke due to firewood used for cooking and warming purposes, high level of smoking, high intake of liquor, and high prevalence of HIV-infection due to large migratory population. After detecting the statistically significant clusters of TB in the region, a survey-based study is intended to identify the role of these factors in the spread of TB. The scope of present study is limited to only one district of Uttaranchal. There is further scope to involve the whole state of Uttaranchal and then the country as a whole. This would mean a critical appraisal of the RNTCP in the whole country.

## Conclusion

The study has shown the presence of three hotspots of TB in Almora district of Uttaranchal, India. Out of these three hotspots detected in the analysis, two are long term whereas one is temporary. The study has also demonstrated that using the existing health data, the spatial scan statistic and GIS can provide public health officials with necessary feedback about the prevalence of statistically significant hotspots of TB in the region, and thus enabling them to chalk out more effective strategy to contain this scourge. More detailed individual level investigations are needed in the identified clusters to ascertain the most important determinants of disease distribution and analyse the burden of TB in this region.

## Methods

### Study area and data collection

The study included the area of Almora district of Uttaranchal, India, situated between 29°32'45.38" and 29°53'57.80" N latitude and 79°14'25.23" and 79°56'7.36" E longitude. Uttaranchal is a newly created hill state of India. According to ancient literature, Uttaranchal is considered abode of Gods. Almora is the cultural capital of Uttaranchal. As per the 2001 census, it has a population of 6,30,444. The literacy rate of the district is 74.53%. Its health needs are mainly catered by the government hospitals. The district has three Community Health Centres (CHCs), 32 Primary Health Centres (PHCs), 51 Allopathic Hospitals, 36 Ayurvedic Hospitals and 5 Homoeopathic Hospitals. There are a total of 60 trained doctors under RNTCP in Almora district. The district is divided into 11 blocks. As per the working units under RNTCP, the district has three tuberculosis units (TU) and 13 microscopic centres (MC). The population distribution of these TUs and MCs and the number of trained workers under RNTCP in the district is given in Table 3. A TU is normally established for a population of 500,000 people at sub-divisional level (Community Health Centre or Taluk Hospital or Block Primary Health Centre). It comprises of a Medical Officer (MOTC), a laboratory supervisor (STLS), and a treatment supervisor (STS). Its role is to supervise RNTCP. An MC is the nodal centre for sputum examination, catering for a population of approximately100,000 people. Under RNTCP, TB patients are entitled for free treatment, only in the MCs falling in the area of their residence. This excludes their possibility of going to another MC for treatment of TB under RNTCP.

**Table 3:** Population-wise distribution of the tuberculosis units (TU) and microscopic centres (MC) in Almora district, India

Sl. No.	T.U.	M.C.	No. of Trained Workers	Population		
				2003	2004	2005
1	Almora	DTCAlmora	51	54908	56281	57688
2		Distt. Hosp. Almora	05	84650	86766	88935
3		Bhasiachhana	23	43499	44586	45701
4		Lamgara	48	12944	13268	13600
5		Dhauladevi	48	4073	41748	42792
6		Takula	15	10019	10269	10526
	T.U. Almora		190	246750	252918	259242
7	Ranikhet	Civil Hosp. Ranikhet	70	88336	90544	92808
8		Someshwar	08	32919	33742	34586
9		Chaukhutiya	43	28422	29133	29861
10		Dwarahat	36	56972	58396	59856
	T.U. Ranikhet		157	206649	211815	217111
11	Bhikiyasen	Bhikiyasen	67	58525	59988	61488
12		Deghat	45	57843	59289	60771
13		Sult	21	60679	62196	63751
	T.U. Bhikiyasen		133	177047	181473	186010
	Total		480	630446	646206	662363

**Figure 4:**
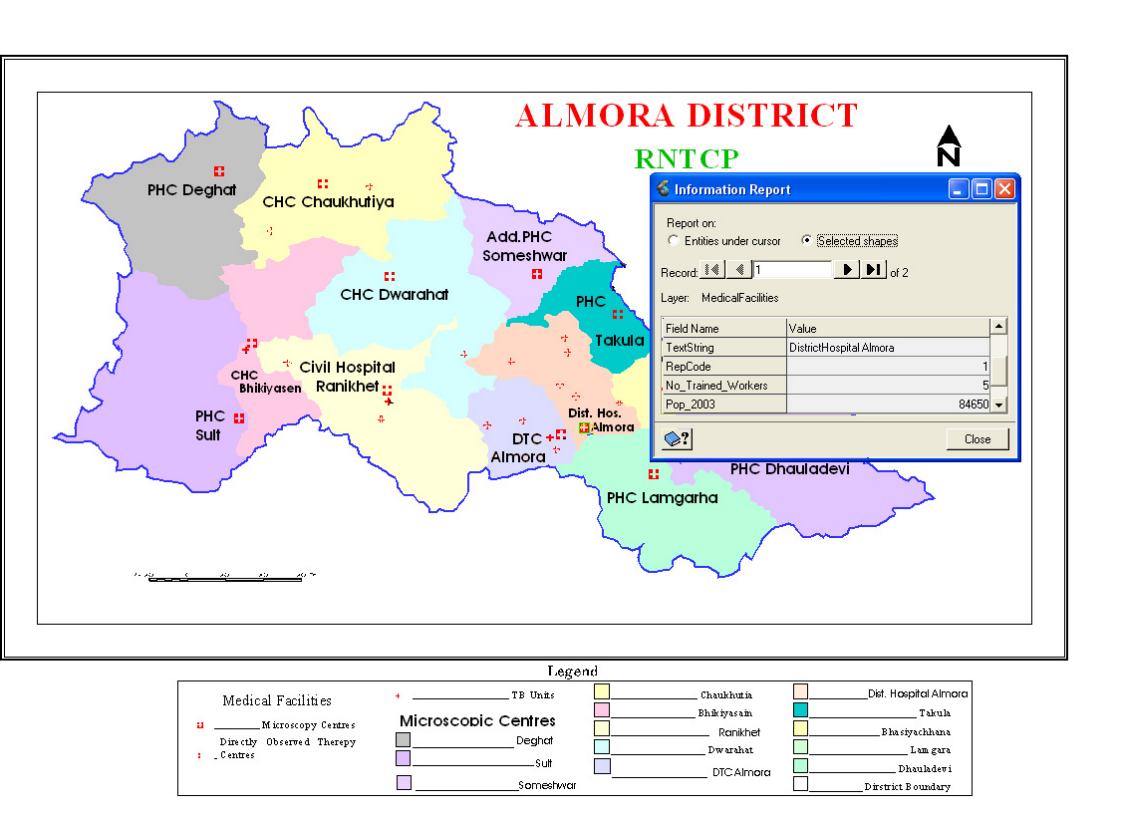
**The thirteen microscopic centres (MC) in Almora district, India with relevant database attached**. This map shows the area covered by the thirteen MCs of Almora district, which are taken as the geographical units of the district for our analyses. The database relevant to these thirteen MCs (Table 3 and Table 4) is also attached to this map.

For the present study, we have collected the secondary data for the tuberculosis cases treated at different hospitals under these thirteen MCs of the district during the year 2003–2005 under RNTCP. The number of TB patients treated under these MCs for the three years are presented in Table 4. For the detection of statistically significant hotspots, we have taken the thirteen MCs of the district as our location points and all the analyses were conducted at the level of these thirteen MCs. The area covered by the thirteen MCs under RNTCP is shown in Figure 4. This map depicts the three TUs, thirteen MCs, and different medical centres as point data in Almora district where the facilities for diagnosis/treatment of TB are available. All the information provided in Table 4 has been fed to point data of MCs (Figure 4) and can be retrieved on a single click of mouse (as shown in figure for District Hospital, Almora). Also, multiple and complex queries can be fixed to reach near to optimal solution of the problem.

**Table 4:** Distribution of tuberculosis (TB) patients in tuberculosis units (TU) and microscopic centres (MC) of Almora district, India

Sl. No.	T.U.	M.C.	Number of TB patients treated		
			2003	2004	2005
1	Almora	DTCAlmora	123	196	136
2		Distt. Hosp. Almora	8	0	0
3		Bhasiachhana	4	14	11
4		Lamgara	22	24	33
5		Dhauladevi	56	42	43
6		Takula	11	8	15
	T.U. Almora		224	284	238
7	Ranikhet	Civil Hosp. Ranikhet	77	93	83
8		Someshwar	24	41	36
9		Chaukhutiya	40	37	41
10		Dwarahat	44	40	40
	T.U. Ranikhet		185	211	200
11	Bhikiyasen	Bhikiyasen	54	51	48
12		Deghat	12	32	25
13		Sult	14	23	34
	T.U. Bhikiyasn		80	106	107
	Total		489	601	545

### Statistical and geographical analysis

#### (a) Detection and identification of tuberculosis clusters

The spatial scan statistic developed by Kulldorff [12] implemented in a software program, SaTScan [13], was used to test the presence of statistically significant spatial as well as space-time clusters of TB and to identify their approximate locations. Purely spatial analysis, which does not take time into account, was performed to detect the TB clusters in the study region of Almora district. The theory behind the spatial scan statistic is the generalization of a test proposed by Turnbull et al. [20]. The spatial scan statistic imposes a circular window on the map and lets the center of the circle move over the area so that at different positions the window includes different sets of neighboring census areas. If the window contains the centroid of the census area, then that whole area is included in the window. For each circle centroid, the radius of the circular window is varied continuously from 0 up to a maximum radius so that the window never includes more than 50% of the total population at risk. The test of significance of the identified clusters is based on a likelihood ratio test [21]. As the likelihood ratio is maximized over all the circles, it identifies the circle that constitutes the most likely cluster. Its p-value is obtained through Monte Carlo hypothesis testing technique proposed by Dwass [44]. If we do a purely spatial analysis for an extensive time period, we have a low power to detect recently emerging clusters. A solution is to use the space-time scan statistic. Instead of circular window in two dimensions, the space-time scan statistic uses a cylindrical window in three dimensions. The base of the cylinder represents space, exactly as in the purely spatial scan statistic, whereas the height represents time. The likelihood ratio test statistic is constructed in the same way as for the purely spatial scan statistic with computational algorithm for calculating the likelihood for each window in three rather than two dimensions.

Identification of spatial and space-time high clusters was done under Poisson probability model assumption with population numbers given in Table 3. The geographical unit of the analysis for the present study is the MC. The purely spatial analyses were performed first, ignoring time. The maximum spatial cluster size was first set to include up to 50% of the population at risks, which included all invasive TB incidence diagnosed between 2003 and 2005. Then it was set at 25% to test for high excesses and to discover smaller, more defined areas of excess. Space-time analyses were then performed to determine whether the clusters of TB obtained from the purely spatial analysis were long term or temporary by taking a spatial window that could include up to 50% of population at risk and a maximum temporal window of 50%, without including purely spatial clusters. The method was then used to check the most likely clusters with 1, 2 or 3-year length clusters. For this, the maximum temporal cluster size was set at 90% of the study period and also included purely spatial clusters with a temporal size of 100%. The maximum spatial cluster size was set at 25% of the population at risk and included purely temporal clusters (spatial size = 100%) as well. Considering the fact that the p-values of the secondary clusters are conservative and therefore they under-estimate their true significance, the significance of secondary clusters was assessed at 10% level of significance whereas the primary clusters was assessed at 5% level. The analyses could not be adjusted for age or any other potential covariates, as the study was based on a hospital-based population and the records of age and socio-economic conditions of the patients were not properly maintained by the hospitals.

#### (b) Geographical analyses

For geographical analysis we have used the techniques available through the Geographical Information System (GIS). Geographical data used were the digital maps from the NRDMS Centre, Almora. All the geographical and cartographic outputs have been presented in Geomatica v10 GIS/RS software [45] having UTM co-ordinate system and information from these maps can be retrieved using any compatible software.

## Competing interests

The author(s) declare that they have no competing interests.

## Authors' contributions

NT was involved in the conceptualisation, research design, execution and write-up of the manuscript. CMSA collaborated in research design and construction of cartographic and GIS part of the study. AT and VK collaborated in research design and conceptualisation of medical and health portion of the study. All authors were involved in the preparation of the manuscript.

## References

[B1] Murray CJL, Lopez AD (1996). The global burden of disease: a comprehensive assessment of mortality and disability from diseases, injuries and risk factors in 1990 and projected to 2020. World Health Organization Document.

[B2] Narain JP, ed (2002). Tuberculosis-epidemiology and control. World Health Organization, Regional Office for South East Asia, New Delhi, India.

[B3] Dye C, Scheele S, Dolin P, Pathania V, Raviglione MC (1999). Global burden of disease: estimated incidence, prevalence, and mortality by country. J Am Med Assoc.

[B4] Directorate General of Health Services TB India 2005: RNTC Status Report. Central TB Division, New Delhi.

[B5] Cauthen GM, Pio A, ten Dam HG (1988). Annual risk of infection. World Health Organization Document.

[B6] Telzak EE (1997). Tuberculosis and Human Immunodeficiency Virus infection. Med Clin North Am.

[B7] World Health Organization (2004). Global Tuberculosis Control-WHO Report.

[B8] Directorate General of Health Services TBC India Template. Central TB Division, New Delhi.

[B9] UNAIDS (2006). Report on global AIDS epidemic.

[B10] Registrar General and Census Commissioner Census of India 2001. http://www.censusindia.net.

[B11] Ministry of Health and Family Welfare National Health Policy 2002. New Delhi, India.

[B12] Kulldorff M (1997). A spatial scan statistic. Communications in Statistics: Theory and Methods.

[B13] Kulldorff M, Information Management Services, Inc. SaTScan™ v6.1 (2006). Software for the spatial and space-time scan statistics. http://www.satscan.org/.

[B14] Wallenstein S (1980). A test for detection of clustering over time. Am J Epidemiol.

[B15] Weinstock MA (1981). A generalized scan statistic test for the detection of clusters. Int J Epidemiol.

[B16] Kulldorff M (2001). Prospective time-periodic geographical disease surveillance using a scan statistic. J R Stat Soc Ser A.

[B17] Michelozzi P, Capon A, Kirchmayer U, Forastiere F, Biggeri A, Barca A, Perucci CA (2002). Adult and childhood leukemia near a high power radio station in Rome, Italy. Am J Epidemiol.

[B18] Viel JF, Arveux P, Baverel J, Cahn JY (2000). Soft-tissue sarcoma and non-Hodgkin's lymphoma clusters around a municipal solid waste incinerator with high dioxinemission levels. Am J Epidemiol.

[B19] Kulldorff M, Athas WF, Feuer EJ, Miller BA, Key CR (1998). Evaluating cluster alarms:A space-time scan statistic and brain cancer in Los Alamos, New Mexico. Am J Public Health.

[B20] Turnbull BW, Iwano EJ, Burnett WS, Howe HL, Clark LC (1990). Monitering for clustersof disease: application to leukemia incidence in upstate New York. Am J Epidemiol.

[B21] Hjalmars U, Kulldorff M, Gustafsson G, Nagarwalla N (1996). Childhood leukaemia in Sweden: using GIS and a spatial scan statistic for cluster detection. Stat Med.

[B22] Sheehan TJ, DeChelo LM (2005). A space-time analysis of the proportion of late stage breast cancer in Massachusetts, 1988 to 1997. Int J Health Georg.

[B23] Cousens S, Smith PG, Ward H, Everington D, Knight RSG (2001). Geographical distribution of varient Creutzfeldt-Jakob disease in Great Britain, 1994–2000. Lancet.

[B24] Chaput EK, Meek JI, Heimer R (2002). Spatial analysis of human granulocytic ehrlichiosis near Lyme, Connecticut. Emerg Infect Dis.

[B25] Sabel CE, Boyle PJ, Loytonen M, Gatrell AC, Jokelainen M (2003). Spatial clustering of amyotrophic lateral sclerosis in Finland at place of birth and place of death. Am J Epidemiol.

[B26] Green C, Hoppa RD, Young TK, Blanchard JF (2003). Geographic analysis of diabetes prevalence in an urban area. Soc Sci Med.

[B27] Odoi A, Martin SW, Michel P, Middleton D, Holt J, Wilson J (2004). Investigation of clusters of giardiasis using GIS and spatial scan statistic. Int J Health Georg.

[B28] Curtis A (1999). Using a spatial filter and a geographic information system to improve rabies surveillance data. Emerg Infect Dis.

[B29] Frank C, Fix A, Peña C, Strickland G (2002). Mapping Lime disease for diagnostic and preventive decisions, Maryland. Emerg Infect Dis.

[B30] Glass GE, Schwartz BS, Morgan JM, Johnson DT, Noy PM, Israel E (1995). Environmental risk factors for Lyme disease identified with geographic information system. Am J Public Health.

[B31] Morrison A, Getis A, Santiago M, Rigau-Perez J, Reiter P (1998). Exploratory space-time analysis of reported dengue cases during an outbreak in Florida, Puerto Rico, 1991–1992. Am J Trop Med Hyg.

[B32] Mott KE, Nuttall I, Desjeux P, Cattand P (1995). New geographical approaches to control of some parasitic zoonoses. Bull World Health Organ.

[B33] Zeman P (1997). Objective assessment of risk maps of tick-borne encephalitis and Lyme borreliosis based on spatial patterns of located cases. Int J Epidemiol.

[B34] Bishai WR, Graham NMH, Harrington S, Pope DS, Hooper N, Astemborski J, Sheely L, Vlahov D, Glass GE, Chaisson RE (1998). Molecular and geographic patterns of tuberculosis transmission after 15 years of Directly Observed Therapy. J Am Med Ass.

[B35] Bifani PJ, Mathema B, Liu Z, Moghazeh SL, Shopsin B, Tempalski B, Driscoll J, Frothingham R, Musser JM, Alcabes P, Kreiswirth BN (1999). Identification of a W variant outbreak of *Mycobacterium tuberculosis *via population based molecular epidemiology. J Am Med Ass.

[B36] Sudre P, Pfyffer GE, Bodmer T, Prod'hom G, Furrer H, Basetti S, Bernasconi E, Matter L, Telenti A, Strässle A, Jacques JP, Weber R (1999). Molecular epidemiology of tuberculosis among HIV-infected persons in Switzerland: A countrywide 9-year cohort study. Infection.

[B37] Antunes JLF, Waldman EA (2001). The impact of AIDS, immigration and housing overcrowding on tuberculosis deaths in São Paulo, Brazil, 1994–1998. Soc Sci Med.

[B38] Indian Council of Medical Research Tuberculosis in India-a sample survey 1955–58. Special Report, Series No 34.

[B39] Chadha VK, Vaidyanathan PS, Jagannatha PS, Unnikrishnan KP, Savanur SJ, Mini PA (2003). Annual risk of tuberculosis infection in the western zone of India. Int J Tuberc Lung Dis.

[B40] Chadha VK, Vaidyanathan PS, Jagannatha PS, Unnikrishnan KP, Mini PA (2002). Annual risk of tuberculosis infection in the northern zone of India. Bull World Health Organ.

[B41] Kolappan C, Gopi PG, Subramani R, Chadha VK, Kumar P, Prasad VV, Appegowda BN, Rao RSN, Shashidharan N, Ganesan N, Santha T, Narayanan PR (2004). Estimation of annual risk of tuberculosis infection among children aged one to nine years in the south zone of India. Int J Tuberc Lung Dis.

[B42] Chadha VK, Kumar P, Gupta J, Jagannatha PS, Magesh V, Singh S, Lakshminarayan, Ahmed J, Srivastava RK, Prasad N, Vaidyanathan PS (2004). Annual risk of tuberculosis infection in the eastern zone of India. Int J Tuberc Lung Dis.

[B43] Comstock GW, Livesay VT, Woolpert SF (1974). The prognosis of a positive tuberculin reaction in childhood and adolescence. Am J Epidemiol.

[B44] Dwass M (1957). Modified randomization tests for non-parametric hypothesis. Ann Math Statist.

[B45] PCI Geomatics Enterprises Inc. Geomatica v10 GIS/RS software.

